# A bacterial NO-binding sensor domain evolved through acquisition of a cytochrome-derived c-type heme-binding motif

**DOI:** 10.64898/2026.02.01.703092

**Published:** 2026-02-02

**Authors:** Maithili Deshpande, Jiawei Xing, Vadim M. Gumerov, Brian R. Crane, Igor B. Zhulin

**Affiliations:** 1 Department of Chemistry and Chemical Biology and The Weill Institute for Cell and Molecular Biology, Cornell University, Ithaca, NY 14853 USA; 2 Department of Microbiology and Translational Data Analytics Institute, The Ohio State University, Columbus, OH, 43210 USA

## Abstract

Diverse bacterial sensory domains detect intracellular and extracellular cues and relay this information to downstream signaling pathways. Per-Arnt-Sim (PAS) domains are well known to bind cofactors such as heme for intracellular sensing, whereas Cache domains - the largest family of bacterial extracytoplasmic sensor modules - are thought to function exclusively as cofactor-independent ligand-binding domains.

Here, we identify and characterize a large family of Cache domains that contain a covalently bound c-type heme. Systematic sequence analysis of the DUF3365/Tll0287 family of unknown function reveals exceptional conservation of a canonical CXXCH heme-binding motif, establishing c-type heme binding as a defining feature of this domain family. Structure- and sequence-based comparisons demonstrate that, despite prior annotation as PAS-like, these domains belong to the Cache superfamily and share a conserved Cache fold augmented by a distinct structural element harboring the heme-binding motif.

Heme containing Cache domains are widely distributed across bacterial phyla, with strong enrichment in Gracilicutes, and frequently occur as stand-alone modules, an atypical presentation of Cache domains. Phylogenetic analyses support insertion of a short cytochrome c–derived fragment containing the heme-binding motif into a preexisting Cache scaffold, representing a previously unrecognized mode of sensor-domain evolution.

Spectral and ligand-binding analyses indicate that heme containing Cache domains fused to signal-transduction modules have properties typical of nitric oxide, but not oxygen, sensors; whereas stand-alone domains likely have redox-related roles. Our findings expand the functional repertoire of Cache domains to include covalently bound cofactors and reveal fragment-level recruitment of enzymatic motifs as a route for the emergence of bacterial sensory systems.

## Introduction

Signal transduction is a fundamental process that enables cells to perceive and adapt to changing conditions. In bacteria, this process involves a diverse set of sensory proteins that detect a wide range of physical and chemical stimuli, including nutrients, toxins, temperature changes, and signaling molecules^1,2^. Upon sensing these cues, these proteins initiate a cascade of intracellular signals, often through reversible phosphorylation or second messenger production, to modulate gene expression, motility, metabolism, and virulence. The largest families of sensory proteins in bacteria include transcriptional regulators^2,3^, histidine kinases^1^, second messenger-based systems, such as those utilizing cyclic di-GMP^4^ or cAMP^5^, and methyl-accepting chemotaxis proteins (MCPs)^6,7^. Collectively, signaling networks employing these sensory proteins enable bacteria to thrive in diverse environments, contributing to their ecological success.

Sensory proteins allow bacteria to detect various cues both intracellularly and externally. These responses are achieved by having dedicated sensor domains that are fused to downstream signaling modules, such as a histidine kinase catalytic domain and MCP signaling domain. Among the many sensor domains that mediate signal detection, Per-Arnt-Sim (PAS) domains are particularly well-studied. PAS domains represent one of the largest superfamilies of intracellular sensory modules that primarily sense redox states, light, and gases, such as oxygen, carbon monoxide (CO), and nitric oxide (NO) by binding cofactors such as flavins or heme^8–10^. By contrast, Cache domains that are homologous and structurally similar to PAS domains are found in the extracytoplasmic regions of sensory proteins, where they function as ligand-binding modules^11,12^. Cache domains form the largest family of extracellular sensors in bacteria^12^ and mediate sensing of external small-molecule ligands, such as amino acids, biogenic amines, nucleotides, organic acids, and sugars^13–17^.

In this study, we describe a large family of Cache sensor domains that evolved through acquisition of a short cytochrome c-derived peptide containing a conserved c-type heme–binding motif. Experimental evidence further demonstrates that this heme c-containing domain fused to downstream signaling modules has properties typical of a nitric oxide sensor. Finally, we show that this sensor is found in many bacterial phyla, with enrichment in Gracilicutes, and it serves as an input to major signal transduction systems in bacteria.

## RESULTS

### The DUF3365/Tll0287 domain family contains a conserved heme c binding motif

Systematic analysis of bacterial signal transduction proteins in the MiST database^18^ revealed that many sensory proteins, including histidine kinases, MCPs, and c-di-GMP metabolizing enzymes harbor an N-terminal domain of unknown function, originally annotated in the Pfam database^19^ as DUF3365 (PF11845). Following the integration of Pfam into the InterPro resource^20^, this domain was renamed Tll0287-like, retaining the same Pfam accession and assigned InterPro accession IPR021796. This domain family was assigned to the PAS_fold superfamily, following the original interpretation by the authors who solved the structure of the *Thermosynechococcus elongatus* protein Tll0287 (PDB: 5B82) and noted that it adopts a fold resembling PAS sensor domains^8,10^ rather than typical c-type cytochrome scaffolds^21^. They further demonstrated that Tll0287 protein contains *c* heme, which is axially ligated by cysteine and histidine residues and harbors a canonical CXXCH motif that forms thioether bonds with the heme vinyl groups^21,22^.

To comprehensively collect DUF3365/Tll0287 sequences from public databases, we employed two complementary approaches. First, we downloaded a curated set of more than 5,000 protein sequences matching the Tll0287-like domain profile from the InterPro database, which contains curated, taxonomically balanced set of genomes (Dataset S1). To explore a larger sequence space beyond this stringently curated dataset, we performed PSI-BLAST searches against the NCBI Clustered NR database^23^ using the Tll0287 protein sequence as a query, which retrieved a larger set of ~20,000 sequences (Dataset S2).

In addition to Tll0287, both datasets included two proteins from *Geobacter sulfurreducens*, GSU0582 and GSU0935, which were previously characterized and structurally resolved. These proteins were assigned to the PAS superfamily and shown to contain covalently bound heme c^24^. We constructed multiple sequence alignments of Datasets 1 and 2, which revealed that the canonical CXXCH c-type heme-binding motif is strictly conserved across this domain family. Conservation rates for this motif in Dataset 1 and 2 were 99% and 99.4%, respectively. Together, these results indicate that covalently bound c-type heme is a defining and likely functionally essential feature of the DUF3365/Tll0287 domain family.

### DUF3365/Tll0287 is a cofactor-containing Cache domain

All prior classifications, including original descriptions of the Tll0287^21^ and GSU0582/GSU0935^24^ proteins, as well as Pfam and InterPro annotations, assigned this domain family to the PAS superfamily. Indeed, PAS domains include several families capable of heme-binding^10^. However, we hypothesized that DUF3365/Tll0287 belonged to the Cache rather than PAS superfamily. This hypothesis was motivated by two observations. First, all previously reported “extracytoplasmic PAS domains” defined based on structural interpretations have subsequently been reclassified as Cache domains following more detailed sequence and structure analyses^12^, Second, bona fide PAS domains that bind heme are cytoplasmic and utilize noncovalently bound heme b^25,26^, whereas c-type heme is invariably located in the extracytoplasmic compartment because heme covalent attachment involves membrane transport^27^.

To re-evaluate the family and superfamily assignment of DUF3355/Tll0287, we performed both structure- and sequence-based searches. In structural comparisons, all ten top matches defined by a DALI^28^ search against the PDB database^29^ using the Tll0287 structure (PDB: 5b82) were annotated as Cache domains by InterPro/Pfam (Table S1). Consistently, profile-profile sequence comparisons using HHpred^30^ also assigned DUF3365/Tll0287 to the Cache superfamily (Table S2). The highest-scoring matches included multiple Cache domain family profiles, as well as PDB profiles corresponding to histidine kinases CitA and DcuS and the MCPs PctA and PctC, all of which are well-established members of the Cache superfamily^12,31^.

**Figure 1.**
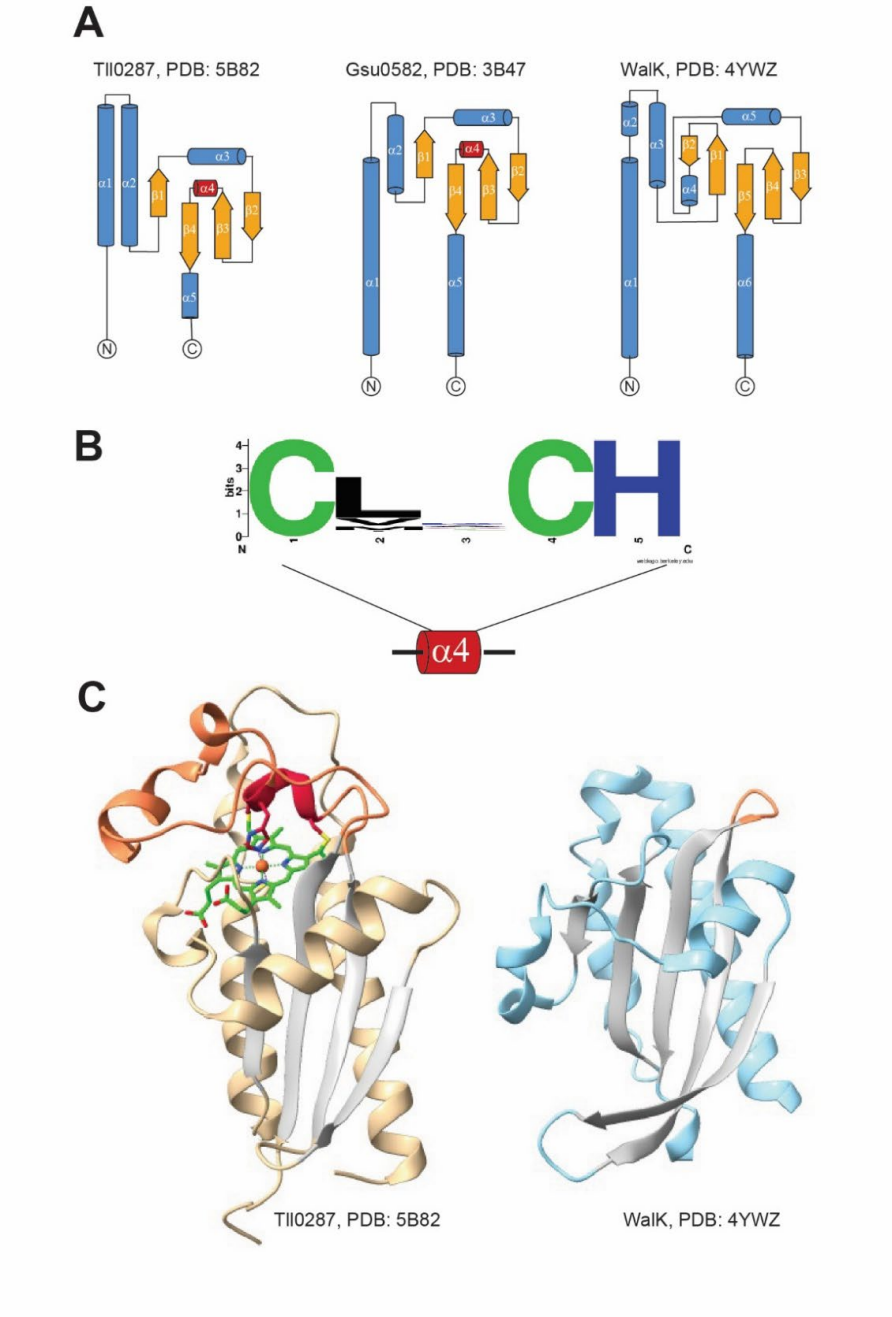
Structural comparison of sCache_heme and sCache domains. **A)** Topology diagrams of periplasmic domains of Tll0287 and Gsu0582 in comparison to the sCache domain of the WalK sensor histidine kinase. Alpha helices are shown in blue and beta strands – in orange. **B**) A short alpha helix (α4) inserted between strands β3 and β4 contains the CXXCH cytochrome c binding site. Sequence logo was generated from a multiple sequence alignment of Dataset 1 (>5,000 sequences). **C**) Side-by-side comparison of Tll0287 and WalK structures. sCache_heme domains have an expanded loop (orange) preceding the last β-strand that contains the heme (green)-binding CXXCH motif (red).

Structurally, the DUF3365/Tll0287 fold shares a conserved core architecture with ligand-binding Cache domains but contains a distinct structural element that is absent from canonical sCache (comprised of a **s**ingle **Cache** module) domains – an insertion between β3 and β4 containing a short α-helix and adjacent loop regions that encompass the CXXCH motif (Fig. 1). Based on these combined sequence and structural features, we designate this domain **sCache_heme**, representing the first known Cache domain that contains a covalently attached cofactor.

### Phyletic distribution and domain architecture of sCache_heme-containing proteins

**Figure 2.**
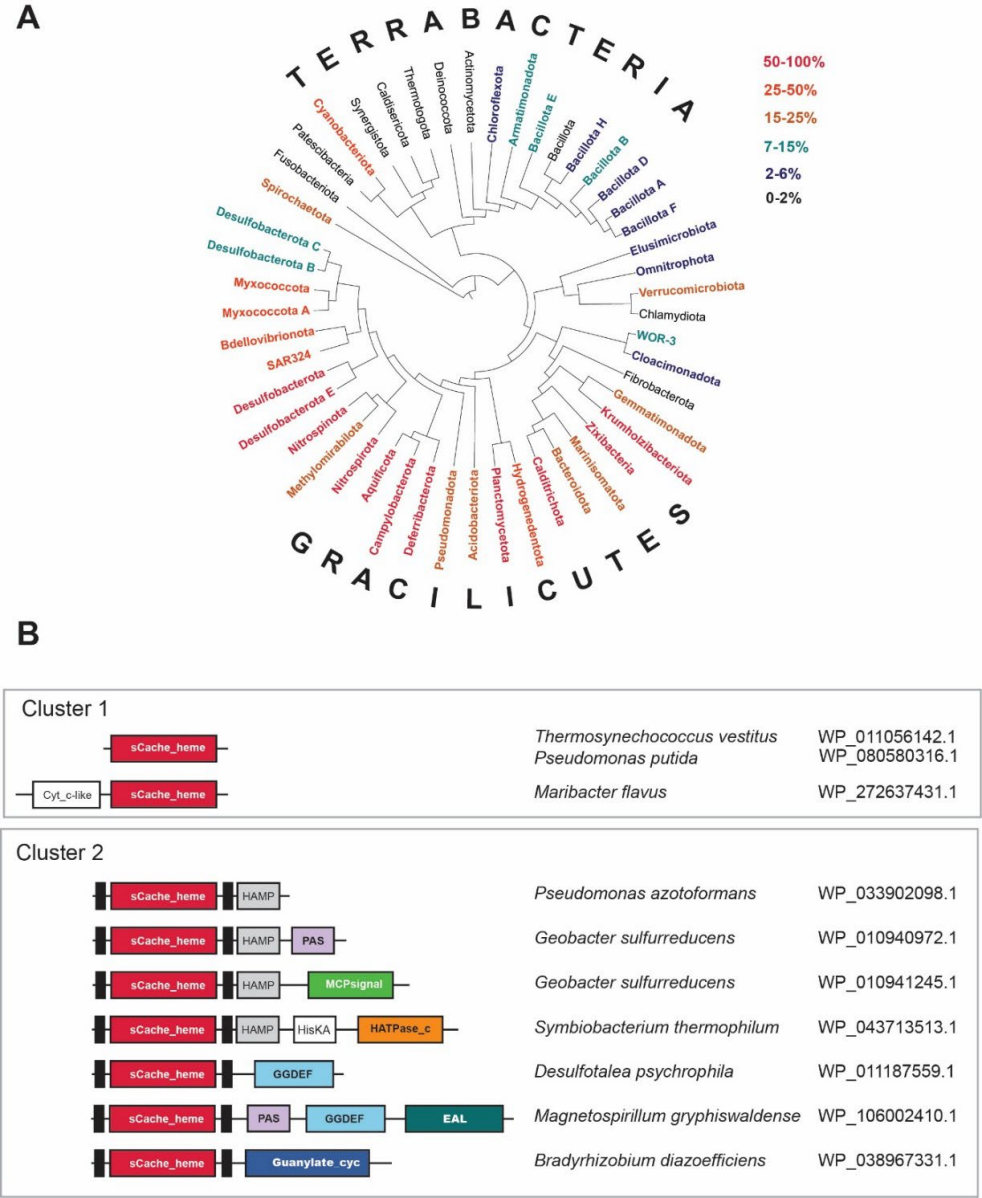
Phyletic distribution and domain architecture of sCache_heme. **A**) GTDB taxonomy tree showing major bacterial phyla. Colors represent the percentage of genomes in a phylum that harbor sCache_heme domain containing proteins. **B**) Most frequent associations of sCache_heme with signaling domains. Representative genomes and accession for sCache_heme proteins are shown. Domain nomenclature according to Pfam: HAMP (PF00672) – present in Histidine kinases, Adenylate cyclases, Methyl-accepting chemotaxis proteins, and Phosphatases; PAS (CL0183) – found in PER, ARNT, and SIM proteins; MCPsignal (PF00015) – Methyl-accepting chemotaxis protein (MCP) signaling domain; HisKA (PF00512) – Histidine Kinase phosphor-acceptor domain; HATPase_c (PF02518) – present in Histidine kinase-, DNA gyrase B-, and HSP90-like ATPase; GGDEF (PF00990) – diguanylate cyclase GGDEF domain; EAL (PF00563) - c-di-GMP phosphodiesterase EAL domain; Guanylate_cyc (PF00211) – Adenylate and guanylate cyclase catalytic domain.

sCache_heme domains are distributed across a broad range of bacterial phyla, but are disproportionately enriched within Cracilicutes, particularly in Defferibacterota, Nitrospirota, Desulfobacterota, Campylobacterota, and Aquificota (Table S3, Fig. 2), where more than 50% of sequenced genomes encode at least one sCache_heme-containing protein. In contrast, these proteins are rare or entirely absent from many Terrabacteria phyla, including Bacillota, Actinomycetota, and Chloroflexota. Cyanobacteriota represents the only Terrabacteria with a substantial fraction of genomes (~30%) encoding sCache_heme (Table S3, Fig. 2). Several deeply branching bacterial phyla – Fusobacteria, Deinococcota, Synergistota, and Thermotogota – are also largely devoid of sCache_heme domains. This phyletic distribution suggests that the sCache_heme emerged within Gracilicutes, followed by limited horizontal transfer into some Terrabacteria lineages.

Analysis of domain architectures revealed that nearly half of all sCache_heme domains occur as stand-alone domains. This organization is highly unusual for Cache family members, more than 90% of which are typically fused to downstream signaling modules such as histidine kinases, MCPs, or GGDEF/EAL enzymes. Notably, a fusion of sCache_heme with a cytochrome_c-like domain (Fig. 2), which also contains the CXXCH motif, was identified exclusively in Flavobacteria. We designated this group as Cluster 1 (Fig. 2). In the remaining cases, sCache_heme domains are fused to classical signaling outputs consistent with their incorporation into modular signal transduction systems. This group was designated as Cluster 2. This distribution raises the question of whether the domains in Cluster 1 also participate in signaling or instead perform redox-related functions similar to cytochromes.

### Origin of the heme c-binding motif in Cache sensor domains

To explore the likely evolutionary scenario underlying heme acquisition by the sCache_heme domain, we performed two complementary sequence similarity searches. First, we conducted BLAST searches with default parameters against the RefSeq and Clustered NR databases, using a 13–amino acid sequence from GSU0582 (WP_010941245.1; PDB: 3B47) - EVRCQSCHEQGAR - corresponding to α-helix 4 and portions of the adjacent loops. In both searches the highest scoring sequences comprised a diverse set of cytochrome family proteins containing the canonical CXXCH motif (Tables S4 and S5).

Second, we performed PSI-BLAST searches (BLASTP lacked sufficient sensitivity) against the RefSeq database using the sCache domain sequence from which the 13-aa region containing the CXXCH motif had been removed. To ensure generality, we carried out this analysis using multiple sCache_heme domains derived from different protein contexts, including MCPs, histidine kinases, and GGDEF/EAL-containing proteins. In addition to sCache_heme domains, these searches consistently retrieved numerous proteins harboring classical sCache domains that lack the CXXCH motif. As an illustrative example, a PSI-BLAST search initiated with the sCache_heme domain of GSU0582 (with the CXXCH-containing segment deleted) retrieved a DcuS/MalK family sensor histidine kinase from *Priestia aryabhattai* (WP_411982187.1). This sequence was recovered in the sixth iteration with a highly significant E value of 3.28 × 10^−17^ (Dataset S3). Its N-terminal region (residues 32–166) yielded the top ten Pfam matches to multiple families within the Cache superfamily (Table S6).

Together, these results support a scenario in which a short fragment derived from a cytochrome c–like protein, carrying the heme-binding CXXCH motif, was inserted into a preexisting stand-alone sCache domain. This interpretation is further supported by the observation that stand-alone sCache_heme domains represent the largest group within this protein subfamily, in contrast to ligand-binding Cache domains reported previously^13–15,32^. In this context, fusions of sCache_heme_heme domains with signal transduction modules leading to the emergence of NO-sensing histidine kinases, MCPs, and cyclic nucleotide–modulating enzymes are likely secondary and more recent evolutionary events.

Recruitment of enzymatic domains as sensors into existing signal transduction pathways is well documented^33,34^. However, to our knowledge, this represents the first example in which only a small fragment of an enzymatic domain was co-opted into a signaling pathway via insertion into an existing sensor domain.

Finally, only a single archaeal genome in the RefSeq database^35^ - *Methanohalophilus levihalophilus* – encodes a sCache_heme containing sensor histidine kinase (WP_209678719.1). A small number of homologs are detectable in archaeal genomes within the larger NR database^23^ (Dataset S2), consistent with the emergence of this domain after the divergence of bacterial and archaeal lineages and with only exceedingly limited horizontal transfer of sCache_heme domains to archaea.

### Target selection for experimental validation

The evolutionary scenario proposed above predicts that sCache_heme domains associated with signal transduction modules (Cluster 2) function as heme-based gas sensors, whereas stand-alone or cytochrome-associated (Cluster 1) may instead serve redox-related roles. To test this hypothesis, we selected two representatives from the phylum *Pseudomonadota*: a stand-alone sCache_heme protein from *Pseudomonas putida* designated as Target 1 (Cluster 1, Fig. 2) and a membrane-associated sCache_heme protein from *P. azotoformans* containing two transmembrane helices and a cytoplasmic HAMP domain designated as Target 2 (Cluster 2). Target 1 is closely related to the canonical Tll0287 protein and was predicted to coordinate heme via Cys/His ligation and exhibit properties consistent with redox functions. Due to the presence of a HAMP domain, which has strictly signaling properties^36,37^, Target 2 was predicted to form a signaling-competent dimer with a more labile heme coordination environment.

### Expression, purification, and heme c incorporation

**Figure 3.**
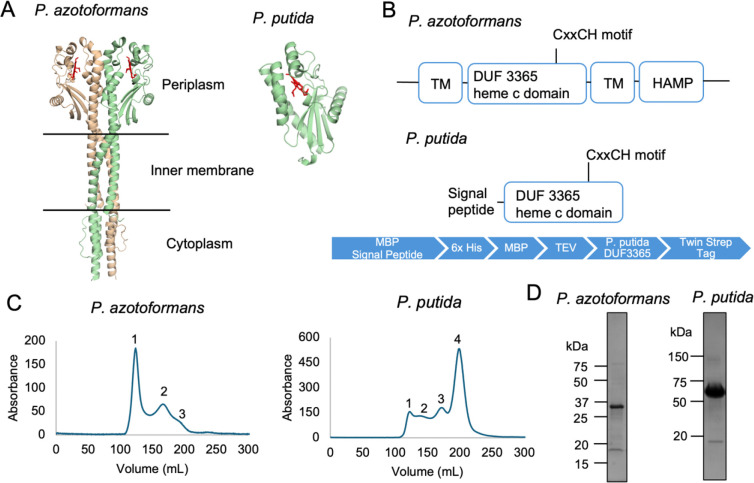
Purification of the sCache_heme domain proteins from *P. azotoformans* and *P. putida.* A. AlphaFold 3 models of the *P. azotoformans* and *P. putida* sCache_heme domain containing proteins. The c-type heme in the periplasmic SCache_heme domain is colored red. The signal peptide for the *P. putida* protein is cleaved during transport to the periplasm and is not present in the structure. B. Domain organization of the sCache_heme proteins. The *P. putida* sCache_heme protein was purified as an MBP-fusion with a TEV protease cleavage site and the expression construct with purification tags is shown in blue. C. Size-exclusion chromatography elution profiles of the sCache_heme proteins on an S200 26/60 column. For the *P. azotoformans* protein, peaks 2 and 3 were pooled for further experiments. The MBP-*P. putida* fusion protein elutes at peak 4. D. Coomassie-stained SDS-PAGE gel of purified *P. azotoformans* and MBP-*P. putida* sCache_heme proteins (34.5 kDa and 64.65 kDa, respectively).

Both sCache_heme proteins were heterologously expressed in *E. coli* by co-expression with the cytochrome c maturation system encoded by plasmid pEC86, which was required for covalent heme attachment. The Target 1 protein from *P. putida* was purified as an MBP-fusion as MBP both greatly improved protein solubility and transported the protein to the periplasm for heme c incorporation. The full-length Target 2 membrane protein from *P. azotoformans* was solubilized in the detergent n-dodecyl-β-D-maltoside (DDM). Both proteins were highly pure (>90% purity) after size-exclusion chromatography (Fig. 3). SEC-SAXS analysis indicated that the Target 2 *P. azotoformans* protein forms a stable dimer in detergent solution (Fig. S1).

### Spectroscopic characterization of redox states

**Figure 4.**
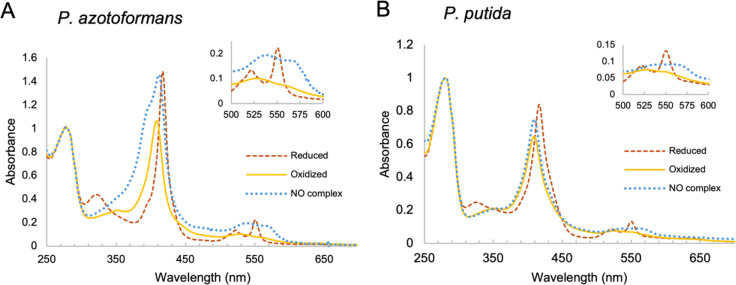
UV-Vis absorption spectra of the *P. azotoformans* and *P. putida* sCache_heme proteins in oxidized (yellow —— ), reduced (red -----) and Fe^2+^-NO forms (blue …….). The insert shows an expanded view of the α and β peaks. Both proteins gave Soret maxima at 417 nm with split α and β bands at ~550 nm and ~521 nm in the Fe^2+^ (reduced) state and Soret maxima at 408 nm with a broad α/β band at ~526 nm in Fe^3+^ (oxidized) state at pH 8.5. The Fe^2+^-NO spectra for *P. putida* sCache_heme showed Soret maxima at 410 nm and relatively flat, diffused α/β peaks with features at ~568 nm and ~530 nm. In contrast, the *P. azotoformans* protein gave spectra with a 412 nm Soret peak and 397 nm shoulder, and distinct α and β bands.

UV-visible absorption spectra of both proteins revealed features characteristic of c-type heme in reduced and oxidized states. (Fig. 4, Table 1). These spectra are consistent with low-spin, six-coordinate heme species. Target 2 lacks the axial Cys residue and was thus predicted to be five-coordinate; however, the low-spin heme spectrum indicates that a water molecule may bind to the heme iron in the 6^th^ coordination position. A Cys-to-Ala substitution (C59A) in the Target 1 *P. putida* protein produced measurable shifts in the Soret and α/β bands, consistent with altered axial ligation (Fig. S2).

The absorption spectra of the two proteins were strikingly different in the Fe^2+^-NO states. The *P. putida* protein formed a six-coordinate Fe^2+^-NO complex with spectral features resembling that of canonical cytochrome c (Table 1), whereas the *P. azotoformans* protein formed a mixture of five- and six-coordinate Fe^2+^–NO species (Fig. 4).

### NO binding and oxygen reactivity distinguishes sensory and redox-like sCache_heme proteins

**Figure 5.**
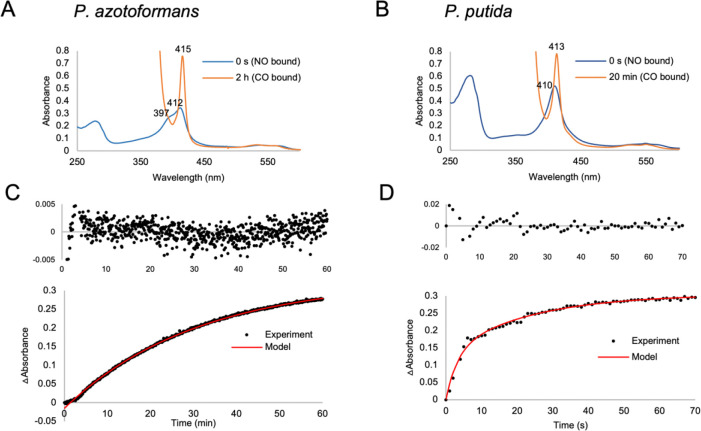
Dissociation of NO from the *P. azotoformans* and *P. putida* Fe^2+^ sCache_heme proteins. A, B. The UV-Vis absorption spectra show conversion of the initial Fe^2+^-NO species (blue) to the final Fe^2+^-CO trap species (orange) upon addition of CO-saturated sodium dithionite buffer into the anaerobic protein solution with a gas-tight syringe. C, D. The increase in absorbance of (C) *P. azotoformans* and (D) *P. putida* CO-bound species was fit to an exponential equation. NO has high affinity for the *P. azotoformans* sCache_heme protein and dissociates slowly over the timescale of one hour, whereas NO dissociates rapidly from the *P. putida* protein within minutes.

Despite similar Fe^3+^ and Fe^2+^ spectra, the two proteins differed markedly in their response to nitric oxide and oxygen. The *P. putida* WT and C59A variants exhibited rapid NO dissociation, indicating weak NO binding (Fig. 5). In contrast, the *P. azotoformans* protein displayed slow NO dissociation, with a k_off_ of (6.25 ± 0.64) × 10^−4^ s^−1^, comparable to established bacterial NO sensors such as NosP and H-NOX (Table 2). NO-induced trans axial ligand dissociation is often a feature of signaling mechanisms by H-NOX proteins. The mixture of five- and six- coordinate NO species formed by the *P. azotoformans* protein has been observed in some cytochrome c’ proteins from photosynthetic bacteria^38–40^, and in NO sensor *Nostoc punctiforme* (*Np*) H-NOX^41^. Unlike *Nostoc punctiforme* H-NOX, where the equilibrium between the two ligation states has a distinct temperature dependence, the mixture of 5- and 6- coordinate species persists in the *P. azotoformans* protein at temperatures of 4, 20 and 40 °C (Fig. S3).

The reduced *P. azotoformans* protein also autoxidizes ~10x faster than the reduced *P. putida* protein, which likely reflects a more exchangeable heme ligand (i.e. water) and accessible distal heme pocket that heighten oxygen reactivity (Fig. S4). Interestingly, the C59A *P. putida* variant does not greatly affect autoxidation rates, thereby suggesting that different degrees of heme accessibility may be the distinguishing factor in oxygen reactivity (Fig. S5). Regardless, none of the *P. putida* WT, *P. putida* C59A or *P. azotoformans* proteins form a stable complex with oxygen, as the *P. azotoformas* protein does with NO (Fig S6). All readily produce superoxide upon reaction with oxygen (Fig. S6). Together, these results demonstrate that sCache_heme domains associated with signal transduction architectures has properties consistent with nitric oxide sensors, but not oxygen sensors, whereas stand-alone sCache_heme domains exhibit properties more consistent with redox-related roles.

## DISCUSSION

In this study, we identify and characterize a previously unrecognized class of Cache sensor domains that bind covalently attached c-type heme and perform distinct biological functions depending on their domain architecture. Through comprehensive sequence analysis, we demonstrate that the DUF3365/Tll0287 family universally contains a highly conserved CXXCH motif, establishing c-type heme binding as a defining and ancestral feature of this domain family. Structure- and sequence profile-based comparisons further resolve earlier ambiguities in domain classification by demonstrating that these proteins belong to the Cache superfamily rather than PAS, underscoring the importance of integrating subcellular localization, cofactor chemistry, and evolutionary context when assigning sensor domains.

Phylogenetic and architectural analyses reveal that sCache_heme domains are broadly distributed across bacterial phyla but show striking differences in domain organization. Approximately half occur as stand-alone domains or in association with cytochrome c–like proteins, whereas the remainder are fused to canonical signal transduction modules such as histidine kinases, MCPs, or cyclic nucleotide enzymes. This dichotomy strongly suggested functional divergence within the family, which we tested experimentally by characterizing representative members from each architectural class.

Biochemical and spectroscopic analyses of sCache_heme proteins from *Pseudomonas putida* (stand-alone) and *P. azotoformans* (signaling-associated) revealed that both proteins bind c-type heme and form low-spin species in reduced and oxidized states. However, despite these similarities, their ligand-binding behaviors differ markedly. The *P. putida* sCache_heme protein, which closely resembles the single-domain protein Tll0287, exhibits stable Cys/His axial ligation and limited ligand exchange, properties consistent with redox-related functions. Indeed, this protein binds neither oxygen nor nitric oxide strongly and undergoes relatively slow autoxidation, consistent with a role in electron transfer or redox sensing.

In contrast, the *P. azotoformans* sCache_heme protein, which is associated with transmembrane helices and a cytoplasmic signal-transmitting HAMP domain, displays properties characteristic of gas-responsive sensors. This protein binds nitric oxide with high affinity while failing to form a stable oxy-complex, a defining feature of selective NO sensors. Its NO dissociation kinetics are comparable to those of established bacterial NO sensors such as NosP and H-NOX, despite its extracytoplasmic localization and covalently bound c-type heme. The formation of mixed five- and six-coordinate Fe^2+^–NO species, together with rapid autoxidation in air, suggests a more labile heme coordination environment that facilitates ligand binding and signal initiation.

Few periplasmic proteins are known to selectively bind NO. Group 1 proteobacterial cytochrome c’ is a rare example of a periplasmic NO binding protein with defined function: NO scavenging for protection against nitrosative stress^42,43^. The Group 1 cytochromes c’ from *Rhodobacter capsulatus* Cc’ (RCCP) has a solvent accessible channel to the distal heme, like the *P. azotoformans* protein, whereas the related group 2 enzymes, such as that from *Alcaligenes xylosoxidans* (AXCP), do not^39^. Interestingly, RCCP forms a mixture of five-and six- coordinate ferrous nitrosyl species with UV-Vis spectra, also very similar to those of the *P. azotoformans* dCache protein, whereas AXCP forms a six-coordinate nitrosyl species like the *P. putida* protein. Thus, the Cluster 2 sCache_heme family members share additional features of NO-reactive proteins.

These findings establish a clear functional division within the sCache_heme family: stand-alone domains exhibit properties consistent with redox-related roles, whereas domains fused to signal transduction modules function as bona fide nitric oxide sensors. This architecture–function relationship parallels, at a conceptual level, the distinction observed among cytoplasmic heme b–binding PAS domains, while revealing an analogous sensing strategy operating in the extracytoplasmic compartment using c-type heme.

From an evolutionary perspective, our results support a model in which a short cytochrome c–derived fragment containing the heme-binding motif was inserted into a preexisting Cache scaffold, giving rise to sCache_heme. Stand-alone sCache_heme domains likely represent the ancestral state, with subsequent fusion to transmembrane and cytoplasmic signaling modules enabling their recruitment into signal transduction pathways. This fragment-level co-option of an enzymatic motif represents a previously unrecognized route for the evolution of sensory domains and expands the functional repertoire of Cache domains to include covalently bound cofactors and gas sensing.

Together, these findings expand the functional repertoire of Cache domains to include covalently bound heme. Such sensors may respond to host-derived NO, denitrification intermediates, or endogenous nitrosative stress, depending on ecological context.

## Materials and Methods

### Bioinformatics tools and databases

MiST 4.0 database^18^ was used for scanning bacterial signal transduction proteins for putative novel sensor domains. The InterPro database^20^ was used to download sequences matching the Tll0287-like domain profile. BLAST and PSI-BLAST searches^44^ against the NCBI Clustered NR^23^ and RefSeq^35^ databases were performed with default parameters. The Pfam database^19^ and the TREND webserver^45^ were used for assessment of protein domain architecture. Structure-based searches against the PDB database^29^ were performed using the DALI server^28^ and profile-profile sequence similarity searches were carried out using HHpred^30^. GTDB (Genome Taxonomy Database) release 10^46^ was used to retrieve taxonomy information and the genome-based, phylum-level phylogenetic tree, which was edited and visualized using iTOL v.6^47^. Multiple sequence alignments were constructed using the L-INS-i algorithm of the MAFFT package (v. 7.490)^48^ and visualized and edited in Jalview v. 2.11.3.064^49^. Sequence logo was generated using WebLogo 3^50^. The AlphaFold Protein Structure Database (AFDB)^51^ was used to retrieve structural models.

### Cloning

Codon-optimized full-length sCache_heme domain-containing proteins from *Pseudomonas putida* and *Pseudomonas azotoformans* (accession numbers WP_080580316 and WP_033902098, respectively) were synthesized by Biomatik (Cambridge, Ontario, Canada) custom gene synthesis service. The *P. putida* DUF 3365 protein lacking its native periplasmic signal-peptide was cloned into pMAL-p2G as an MBP-fusion protein with an N-terminal 6x His tag, TEV protease cleavage site and C-terminal twin-Strep tag using kinase, ligase and Dpn1 (KLD) cloning. The *P. azotoformans* DUF 3365 protein was cloned into pET28a with an N-terminal 6x His tag between restriction enzyme sites Nde1 and Xho1 using Gibson assembly. The *P. putida* C59A variant protein was generated by QuikChange site-directed mutagenesis. All plasmids were confirmed by DNA sequencing at the Cornell University Biotechnology Center.

### Protein expression and purification

#### Purification of the P. azotoformans sCache_heme protein

The *P. azotoformans* FL DUF 3365 gene in pET28a was co-expressed with plasmid pEC86 in *E. coli* BL21 (DE3). pEC86 encodes eight cytochrome c (Cc) maturation proteins that are required for the transport and covalent attachment of heme to apo c-type heme proteins^52^. Cells were grown in Terrific broth (IBI Scientific) containing 25 μg/mL chloramphenicol and 50 μg/mL kanamycin at 37°C to an OD 600 of ~0.8 and induced with 0.5 mM IPTG at 18°C overnight. Cultures were supplemented with 25 μg/mL 5-aminolevulinic acid (Chem-Impex International) at an OD 600 of ~0.4 to increase heme synthesis. Cells were harvested after ~16 hr by centrifugation at 4000 × g for 15 min at 4 °C.

To isolate membranes, cells were resuspended in lysis buffer (25 mM Tris pH 8.5, 500 mM NaCl, 10% glycerol), incubated with 0.25 mg/mL lysozyme and lysed using an Avanti Emulsiflex C3 high pressure homogenizer. Membrane fractions were prepared by resuspending the pellet in lysis buffer after ultra-centrifugation of the lysate at 150,000 × g. Protein was solubilized by incubating membranes in lysis buffer supplemented with 1% n-dodecyl-β-D-maltoside (DDM) (Chem-Impex International) for 90 min at 4°C. Insoluble material was pelleted by centrifugation at 75,000 × g for 1 hr. The clarified supernatant was incubated with Ni-NTA resin for 1 hr and washed with 20 column volumes of wash buffer (25 mM Tris pH 8.5, 500 mM NaCl, 10% glycerol, 30 mM imidazole, 0.1% DDM). Protein was eluted in elution buffer (25 mM Tris pH 8.5, 500 mM NaCl, 10% glycerol, 300 mM imidazole, 0.1% DDM) and then quickly desalted into desalt/GF buffer (25 mM Tris pH 8.5, 250 mM NaCl, 5% glycerol, 0.03% DDM) to remove imidazole. The desalted protein was then run over an S200 26/60 gel filtration column pre-equilibrated with desalt/GF buffer. The protein was stored at 4°C and used within 72 hr.

#### Purification of the P. putida sCache_heme protein

The MBP-*P. putida* DUF 3365 fusion protein in pMAL-p2G was co-expressed with plasmid pEC86 in *E. coli* BL21 (DE3). Cells were grown in Luria-Bertani broth (Difco) containing 25 μg/mL chloramphenicol and 100 μg/mL ampicillin to an OD 600 of ~0.3. Cultures were then supplemented with 25 μg/mL 5-aminolevulinic acid and induced with 0.5 mM IPTG at an OD 600 of ~0.6. Cells were harvested after ~16 hr by centrifugation at 4000 × g for 15 min at 4°C. Cells were resuspended in 25 mM Tris pH 8.5, 500 mM NaCl, 10% glycerol and lysed by homogenization. Lysate was centrifuged at 75,000 × g for 1 hr and clarified lysate was incubated with Strep-Tactin XT 4Flow resin (IBA Lifesciences) for 1 hr at 4°C. Resin was washed with 15 column volumes of lysis buffer and protein was eluted in 25 mM Tris pH 8.5, 500 mM NaCl, 10% glycerol, 50 mM D-biotin. The protein was then run over an S200 26/60 gel filtration column pre-equilibrated with GF buffer (25 mM Tris pH 8.5, 250 mM NaCl, 5% glycerol). Fractions containing protein were concentrated, flash-frozen in liquid nitrogen and stored at −80°C.

### Size-exclusion chromatography coupled small-angle x-ray scattering (SEC-SAXS)

SEC-SAXS experiments on the *P. azotoformans* sCache_heme protein were performed at the Cornell High Energy Synchrotron Source (CHESS). Protein sample at a concentration of 3 mg/mL was loaded onto a Superdex 200 5/150 GL column (Cytiva) at 4°C. Sample was eluted at a flow rate of 0.1 ml/min and the elution flowed directly into the x-ray sample cell. The X-ray flux was 7.51 × 10^12^ photons/s with an energy of 11.35 keV and wavelength of 1.094 Å. The sample-detector distance was 1781 mm. Exposures were collected every 2 s throughout the elution until the profile had returned to buffer baseline. The data was analyzed using BioXTAS RAW^53^ **a**nd Guinier and Kratky analysis was performed.

### Autoxidation assays

The *P. azotoformans* sCache_heme protein or MBP-*P. putida* fusion protein (WT or C59A variant) was degassed overnight in an anaerobic chamber (Coy Laboratory Products) on a 4°C dry bath (Benchmark Scientific). Samples were incubated with a 5x molar excess of potassium ferricyanide for 1 hr to oxidize the heme. Ferricyanide was removed by using a 7k MWCO Zeba spin desalting column (Thermo Scientific). Protein containing Fe^2+^-unligated heme was prepared by incubating with 1 mM dithionite for 1 hr followed by desalting to remove dithionite. To study O_2_ binding, 1.8 mL of 12 μM protein was transferred to a quartz septum-sealed cuvette with a stir bar in an anaerobic chamber and then removed to ambient conditions. UV-Vis spectra were recorded on an Agilent 8543 spectrophotometer for 30 min, stirring at 600 rpm at 20°C. Time traces were initiated by opening the lid of the cuvette and exposing the protein to air. Experiments were done in triplicate. For comparison purposes, rates of oxidation were calculated by fitting the decay in the reduced Soret peak to an exponential decay (y = a^−bx^) after the initial mixing lag time associated with exposing the sample to air.

### Determination of k_off_ for NO complexes

Fe^2+^-unligated protein was prepared as described above. To prepare NO complexes, protein was incubated with a 10x molar excess of nitric oxide donor DEA NONOate (Cayman Chemical). DEA NONOate was then removed by desalting. A CO-dithionite trap was used to measure NO dissociation rate constants. CO was bubbled through degassed buffer containing sodium dithionite. To 1.8 mL of 10 μM protein in an anaerobic septum-sealed cuvette, 200 uL of CO-dithionite solution was injected using a gas-tight syringe to a final dithionite concentration of 30 mM. UV-Vis absorption spectra were recorded over time at 20°C. Dissociation of NO was measured through the decrease in the CO peak (413 nm for MBP-*P. putida* sCache_heme fusion and 415 nm for the *P. azotoformans* FL sCache_heme protein). Experiments were done in triplicate. The change in absorbance was fit to the exponential equation y = a(1 - e^−bt^) + c(1 - e^−dt^) for the MBP-*P. putida* fusion protein and to y = a(1 - e^−bt^) + c for the *P. azotoformans* protein.

### Reactive oxygen species assay

To determine if the proteins were re-oxidizing to the unliganded Fe^3+^ state or the liganded Fe^2+^-O_2_ during the oxygen oxidation experiments, superoxide production was indirectly monitored using the Amplex Red Hydrogen Peroxide/Peroxidase Assay kit (Invitrogen). The kit detects hydrogen peroxide generated by spontaneous dismutation of superoxide to H_2_O_2_. A standard curve was generated using 0–10 μM of H_2_O_2_. The assay was performed in triplicate using 1, 10 and 40 μM of unligated Fe^2+^ protein in a reaction volume of 100 uL in a 96-well plate. The amount of H_2_O_2_ generated due to superoxide release during oxidation on exposing samples to air was hence determined by the reaction of Amplex red with horse radish peroxidase (HRP) via the manufacturer’s specifications.

## Supplementary Material

Supplement 1

Supplement 2

Supplement 3

Supplement 4

Supplement 5

Supplement 6

Supplement 7

Supplement 8

Supplement 9

Supplement 10

## Figures and Tables

**Table 1.** Soret, α and β peaks of the sCache_heme proteins, c-type heme proteins, and NO sensors

	Soret (nm)	β (nm)	α (nm)	Reference
**Fe^2+^**				
*P.azotoformans* sCache_heme	417	522	551	this work
*P. putida* sCache_heme	417	521	550	this work
Cytochrome c (pH 6.8)	415	521	550	^54^
*Rc* cytochrome c’ (RCCP)	425/435	550		^39^
GSU0582	415	522	551	^24^
GSU0935	415	522	550	^24^
sGC	431	555	555	^55^
*Pa* NosP	420	524	554	^56^
*Tt* H-NOX	431	565		^57^
**Fe^3+^**				
*P.azotoformans* sCache_heme	408	528		this work
*P. putida* sCache_heme	408	526		this work
Cytochrome c (pH 6.8)	409	530		^54^
GSU0582 (physiological pH)	401			^24^
GSU0582 (alkaline pH)	410			^24^
GSU0935 (alkaline pH)	408			^24^
**Fe^2+^-NO**				
*P.azotoformans* sCache_heme	397/412	538	563	this work
*P. putida* sCache_heme	410	560 (diffused)		this work
Cytochrome c (pH 13)	411	538	567	^54^
*Rc* cytochrome c’ (RCCP)	395/414	~540	~570	^39^
*Ax* cytochrome c’ (AXCP)	415	540	575	^38^
sGC	398	537	571	^58^
*Pa* NosP	396	534	574	^56^
*Tt* H-NOX	420	547	575	^57^
**Fe^2+^-CO**				
*P.azotoformans* sCache_heme	415	533	560	this work
*P. putida* sCache_heme	413	529	550	this work
Cytochrome c (pH 14)	415	533	560	^54^
*Rc* cytochrome c’ (RCCP)	417	535	566	^39^
sGC	423	541	567	^58^
*Pa* NosP	416	538	565	^56^
*Tt* H-NOX	424	544	565	^57^

**Table 2.** NO dissociation rate constants

	k_1_ (s^−1^)	k_2_ (s^−1^)	Reference
*P. azotoformans*	(6.25 ± 0.64) × 10^−4^	NA	this work
*P. putida*	(6.11 ± 1.54) × 10^−2^	(5.73 ± 2.86) × 10^−1^	this work
*sGC*	(3.6 ± 0.8) × 10^−4^	NA	^58^
*SwH-NOX*	(15.2 ± 3.5) × 10^−4^	NA	^59^
*NosP*	(1.8 ± 0.5) × 10^−4^	NA	^56^
